# The diagnostic conundrum of late-onset developmental regression in child psychiatry: case series

**DOI:** 10.1192/bjo.2024.840

**Published:** 2025-01-27

**Authors:** Shalu Elizabeth Abraham, Sakhardande Kasturi Atmaram, Poornima Khadanga, Nirmalya Mukherjee, Rajendra Kiragasur Madegowda, Harshini Manohar

**Affiliations:** Department of Child and Adolescent Psychiatry, National Institute of Mental Health and Neurosciences, Bangalore, India

**Keywords:** Developmental regression, childhood disintegrative disorder, late-onset regression, childhood-onset schizophrenia, autistic regression

## Abstract

**Background:**

Developmental regression in children, in the absence of neurological damage or trauma, presents a significant diagnostic challenge. The complexity is further compounded when it is associated with psychotic symptoms.

**Method:**

We discuss a case series of ten children aged 6–10 years, with neurotypical development, presenting with late-onset developmental regression (>6 years of age), their clinical course and outcome at 1 year. A comprehensive clinical evaluation, laboratory investigations and neuroimaging ruled out any identifiable neurological cause.

**Results:**

Mean age at regression was 7.65 (s.d. 1.5) years and mean illness duration was 10.1 (s.d. 8.5) months. The symptom domains included regression (in more than two domains – cognitive, socio-emotional, language, bowel and bladder incontinence), emotional disturbances, and hallucinatory and repetitive behaviours. Response to treatment was gradual over 6 months to 1 year. At 1-year follow-up, nine children did not attain pre-regression functioning, and residual symptoms included not attaining age-appropriate speech and language, socio-emotional reciprocity and cognitive abilities.

**Conclusions:**

These cases demonstrate a unique pattern of regression with psychiatric manifestations, distinct from autism spectrum disorder and childhood-onset schizophrenia. The diagnostic dilemma arises from the overlap of symptoms with childhood disintegrative disorder (CDD), childhood-onset schizophrenia and autism. This study underscores the diagnostic intricacies of this clinical presentation and highlights the need for longitudinal follow-up to unravel the transitions in phenomenology, course and outcome. For severe manifestations such as developmental regression, where the illness is still evolving, considering CDD as a non-aetiological and transitory/tentative diagnosis would aid against premature diagnostic categorisation and provide scope for ongoing aetiological search.

The progressive loss of previously acquired developmental milestones and competencies is a concerning clinical picture in child psychiatry and neurology, posing diagnostic and intervention challenges. It may suggest an underlying neurological aetiology denoted by the term ‘progressive intellectual and neurological deterioration’.^1^ There is evidence for developmental regression, particularly early regression during the first 2 years of life in autism spectrum disorder (ASD), epileptic encephalopathies, genetic syndromes such as Rett's syndrome and Phelan McDermid syndrome, and late regression in Down syndrome.^2–4^ Apparent developmental and behavioural regression is observed in childhood-onset schizophrenia (COS)^5–7^ and post-traumatic stress disorder,^1,8^ although there is a paucity of systematic literature on this phenomenon.^6^

The National Institute of Mental Health COS longitudinal study, the largest study of COS to date, reported a prevalence of 0.04%.^9–11^ COS (onset <13 years) is rare in occurrence, and symptom dimensions include both positive and negative symptoms, with disorganisation being less common.^12^ Developmental deviance, especially in the speech and language domains, and poor premorbid adjustment before illness onset have been reported in COS.^13,14^ The diagnosis of psychotic symptoms in children is often complex because of the symptomatic overlap with other emotional, behavioural and neurodevelopmental disorders. When psychotic symptoms are associated with developmental regression, the diagnostic dilemma becomes far more pronounced. Depression in pre-pubertal children, especially among children with atypical neurodevelopment with severe psychomotor retardation, catatonic symptoms and hallucinatory behaviours is an important differential.^15,16^

Regression in the socio-communication domain is commonly seen in ASD, termed ‘autistic regression’.^17^ Meta-analytic reviews report prevalence rates of 30–32.2% of regression in ASD, with an average age at onset of 19.8 months.^18,19^ Prospective designs may have better potential to capture more subtle skill loss, particularly early socio-communication behaviours, compared with retrospective studies.^20^

The phenomenon of autistic regression does not explain the later age of onset at regression, which occurs in a subset of children. Theodore Heller first described this phenomenon ‘*dementia infantalis’* in six previously normal children who presented with developmental regression.^21^ In the ICD-10 and DSM-IV-TR, the disorder was termed childhood disintegrative disorder (CDD).^21^ Misdiagnosis of CDD as COS, potentially attributable to the severe social impairment and withdrawn behaviour accompanied by stereotypic movements resembling symptoms of psychosis, have been reported.^22^ Also, psychotic symptoms were observed in about 33% of children who received a diagnosis of CDD.^21,23,24^

## Diagnostic conundrums in late-onset developmental regression

With the advent of technology, causes for later-onset developmental regression, such as autoimmune encephalitis, neurometabolic and neurodegenerative disorders, were increasingly identified. CDD as a distinct diagnosis has been a subject of debate because of its shared characteristics with ASD, such as core socio-communication impairments, comorbid intellectual disability and epilepsy; thus, CDD was subsumed under ASD. Although the debate on the diagnostic validity continues, CDD does have features supporting it as distinct from ASD.^23–25^ Despite increasing scientific and clinical interest, developmental regression continues to pose multiple challenges. Atypical to what is currently understood about this phenomenon, a subset of children continue to present with later-onset regression in the background of neurotypical development, with no identifiable neurological causes.

This paper describes a large case series of ten children who presented with late-onset developmental regression (>6 years of age) in the background of neurotypical development, their phenomenology, clinical course and outcome at 1-year follow-up. Similar presentations have been reported earlier; however, the age at onset of regression was much earlier (mean age 36 months), with gaps in information on complete aetiological evaluation and longitudinal course and outcome.^21,24^ The paper discusses key considerations for CDD as a ‘transitory’ and ‘non-aetiological’ diagnosis, and the need for longitudinal evaluation for a more reliable diagnostic ascertainment in this subset of children.

## Method

The study was conducted at the National Institute of Mental Health and Neurosciences, a tertiary care teaching hospital at Bangalore, India. Children presenting with late-onset developmental regression (>6 years) with neurotypical development before regression were studied. Ten children fulfilling the eligibility criteria were identified over a period of 5 years (January 2019 to December 2023). Nine out of ten children were evaluated and treated as in-patients, and were prospectively followed up at 1, 3 and 6 months and 1 year since initial presentation. We reviewed the medical records to collect clinical details, evaluation, prospective clinical follow-up of developmental trajectory, course and outcome. This is a case series, based on hospital record review.

### Ethics statement and approval

The study was approved by the Institute Review Board (IRB) of the National Institute of Mental Health and Neurosciences (approval number NIMHANS/IEC (BEH. SC.DIV.) 2024) (IRB proposal and approval: see Supplementary Files 1 and 2 available at https://doi.org/10.1192/bjo.2024.840) for preparing this case series using information available exclusively from clinical records stored at the hospital. Informed consent was obtained from parents of all children who were part of this study. Written informed consent was obtained from four participants and verbal consent was obtained from the remaining six participants, which was witnessed and formally recorded (informed consent form: Supplementary File 3). Given the nature of illness, the children were not in a position to provide informed assent (IRB approval: Supplementary File 2)

Our sample included six males and four females, with a mean age of 8.3 (s.d. 1.7) years. Mean age at regression was 7.65 (s.d. 1.5) years (range: 6–9.5) and mean illness duration was 10.1 (s.d. 8.5) months (range: 1–24). All children were comprehensively evaluated by paediatric neurologists, and possible organic causes were ruled out through extensive investigations (Table 2). None of the children had motor regression, seizures, abnormal neurological signs or sensory impairment, as per clinical evaluation.

Ascertainment of developmental regression was done through the following methods (a) parent report of pre-regression developmental competencies and corroborative information from extended family members and school teachers; (b) serial observation of developmental competencies; (c) a comparative assessment of socio-adaptive functioning pre-regression, after the onset of regression and at follow-up time points; and (d) pre-regression videos of play and dyadic interaction of the child for supportive information regarding the pre-regression developmental competencies.^4^

## Results

### Case descriptions

**Fig. 1:**
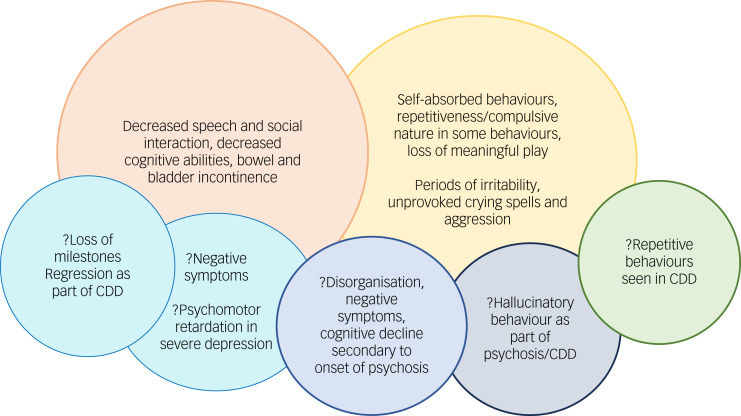
Diagnostic conundrum of developmental regression in children. CDD, childhood disintegrative disorder.

Details of clinical histories and initial presentations are described below. Phenomenological indicators (Table 1) (Fig. 1), treatment details, course and outcome, laboratory and neuroimaging investigations (Table 2) are tabulated. All children had uneventful perinatal history, typical neurodevelopment and had attained age-appropriate milestones before illness onset.

**Table 1:** Comparison of phenomenology and symptom dimensions of the ten cases

Case	Regression of milestones in more than two developmental domains (speech, social, cognitive)	Bowel and bladder incontinence	Hallucinatory-like behaviour (at any point during the illness course)	Delusions	Disorganisation (speech/thought/behaviour)	Flat/blunted affect/negative symptoms	Repetitive and stereotypical behaviours	Emotional disturbances such as crying spells, mood dysregulation, aggression	Comorbid psychiatric or developmental disorder	Catatonic symptoms
Case 1	+	+	+	−	+/−	+/−	+	+	+/-	-
Case 2	+	+	+	−	+/−	+/−	+	+	+^b^	−
Case 3	+	+	+	+/−	+/−	+/−	+	+	+/−	−
Case 4	+	−^a^	+	−	+/−	+/−	+	+	−	−
Case 5	+	+	+	−	+/−	+/−	+	+	−	−
Case 6	+	+	+/−	+/−	+/−	+/−	+/-	+	−	−
Case 7	+	+	+	+/−	+/−	+/−	+	+	−	−
Case 8	+	+	+	−	+/−	+/−	+	+	−	−
Case 9	+	−	+	−	+/−	−	+	+	−	−
Case 10	+	−^a^	+	−	+/−	+/−	+	+	−	−

+, present; −, absent; +/−, cannot be commented on.

a. No incontinence but needed prompts and assistance for all activities of daily living, from being independent in activities of daily living before illness onset.

b. Obsessive–compulsive disorder was diagnosed with reasonable certainty only in one case.

**Table 2:** Clinical profile, biochemical and neuroimaging investigations, course and outcome

Case	Demographic profile, perinatal and developmental history	Family history	Illness duration at initial assessment	Age at onset of regression	Evaluation of neurometabolic disorders^a^, MRI brain, EEG, autoimmune profile	Pharmacotherapy	Course and outcome		Areas of improvement	Residual symptom domains
							3 months	6 months to 1 year		
1	6-year-old male. Uneventful perinatal history, normal development until 6 years	Psychotic illness and completed suicide in third-degree relative	2 months	6 years	Within normal limits	Risperidone 0.5 mg, fluoxetine 10 mg	Decreased hallucinatory behaviour and emotional disturbances, improved speech output, play and academic engagement	Complete remission of hallucinatory behaviour and emotional disturbances, improvement in speech, play, engagement in school activity independent self-care. Slowness in activities and need for prompts for social engagement	Hallucinatory behaviours and emotional dysregulation	Socio-emotional reciprocity, cognitive and ADLs
2	8-year-old female. Uneventful perinatal history, normal development until 6 years	Psychotic illness in third-degree relative	2 years	6.5 years	Within normal limits	Risperidone 3 mg, sertraline 200 mg	Decreased hallucinatory behaviour and obsessive–compulsive, symptoms but persisting irrelevant talk, needs prompts for daily activities	Fluctuating course of hallucinatory behaviour and obsessive–compulsive symptoms, continues to have speech and social difficulties. Dyadic play behaviours improved, but not age-appropriate	Hallucinatory behaviour, obsessive–compulsive phenomenon	Speech and language impairment, socio-emotional reciprocity and play
3	9-year-old female. Uneventful perinatal history, normal development until 9 years	Schizophrenia in third-degree relatives	2 months	9 year	Within normal limits. In addition, cerebrospinal fluid NMDA–negative ANA, ANCA–negative, C–reactive protein, rheumatoid factor-negative, serum ceruloplasmin and copper – normal	Aripiprazole up to 20 mg (minimal response), hence changed to risperidone up to 3 mg (positive response), fluoxetine 10 mg	Minimal improvement	Improvement in hallucinatory behaviour, better social interaction and speech, improvement in self-care after initiating risperidone, but continues to have decreased initiative in peer interaction or play	Hallucinatory behaviour, ADLs	Speech and language, socio-emotional engagement and reciprocity, play
4	6-year-old male. Uneventful perinatal history, normal development until 6 years	Nil	1 month	6 years	Within normal limits	Risperidone up to 3 mg	Significant improvement in psychotic symptoms, restlessness, and anger out bursts, but continues to have self-absorbed behaviour, occasional talking to self, lack of peer interaction. ADLs need prompts and assistance	Improvement in psychotic symptoms, restarted school but does not stay for more than 2 h, plays with sister cooperatively under supervision. Obeys instructions, does all ADLs without any prompts	Hallucinatory behaviour, ADLs	Speech and language, cognitive and socio-emotional reciprocity
5	10-year-old male. Uneventful perinatal history, normal development until 10 years.	Nil	1 year	9.5 years	Within normal limits. In addition, copper profile and ANCA were normal	Risperidone uptitrated to 7 mg over a year, sertraline up to 25 mg for anxiety symptoms	Improvement in expressive language (speaks four- to five-word sentences, narrates stories, engages in meaningful conversation), attends school, improvement in reading and writing, ADLs independent. However, continues to have hallucinatory behaviour when alone	Improvement in language and cognitive domains, but not age-appropriate. Improvement maintained in ADLs, but hallucinatory behaviour and self-absorbed behaviour present when unengaged	ADLs	Language, cognitive domains. Hallucinatory behaviour
6	10-year-old female. Uneventful perinatal history, normal development until 10 years	Schizophrenia in father	1 year	9 years	Within normal limits	Risperidone up to 8 mg/day, lorazepam up to 6 mg/day, augmentation with haloperidol up to 10 mg/day, propranolol up to 40 mg/day (for activity levels)	Improvement in aggression, bowel and bladder incontinence, speech output and interactions, significant weight gain and increased appetite	Fluctuating course at 1 year. Re-emergence of incontinence, restricted repertoire of activities, communication limited to need-based, episodes of aggression continues to persist. aggression, decreased initiative, a sociality, amotivation	–	Speech and language, cognitive, socio-emotional reciprocity, aggression
7	7-year-old female. Uneventful perinatal history, normal development until 7 years	Obsessive–compulsive personality traits in father	6 months	6.5 years	Within normal limits	Aripiprazole 5 mg	Improvement in hallucinatory behaviour and ADLs. Persistent symptoms are amotivation, poor peer interaction	Improved socio-emotional responsiveness with adults, but not with peers. Schooling continued with mother's assistance, gradual improvement in academic and cognitive aspects. Hallucinatory behaviour persistent	Cognitive domains	Hallucinatory behaviours, socio-emotional responsivity
8	7-year-old male. Uneventful perinatal history, normal development until 6 years	nil	6 months	6.5 years	Within normal limits	Risperidone 6 mg, sertraline 17.5 mg, Clonidine 50 μg	Significant improvement in hallucinatory behaviour and anger outbursts. Restarted school, good engagement in age-appropriate play	Relapse of symptoms when sertraline was discontinued. On restarting, improvement in anger outbursts and biological functions noted, although residual self-absorbed behaviours were present intermittently	Socio-emotional reciprocity and responsiveness, emotional problems	Hallucinatory behaviours, speech and language
9	10-year-old male. Uneventful perinatal history, normal development until 8 years	Nil	2 years	8 years	Within normal limits	Risperidone up to 1 mg over a period of 3 months	Minimal improvement in self-absorbed behaviours noted with risperidone, with no improvement in other domains	Gradual improvement in self-absorbed behaviours and expressive language, but did not attain premorbid functioning	Self-absorbed behaviours, emotional disturbances	Speech and language, socio-emotional and cognitive domains
10	10-year-old male. Uneventful perinatal history, normal development until 10 years	Depression in mother, parents separated and maternal grandparents were primary caregivers	1 year	9.5 years	Within normal limits	Escitalopram 10 mg, risperidone 4 mg	Minimal improvement in self-absorbed behaviours	Gradual improvement in expressive language abilities noted. However, self-absorbed behaviours and socio-communication continues to be impaired	Expressive language	Hallucinatory and play behaviours, socio-communication

Autoimmune encephalitis panel: anti-NMDA, anti-N-methyl-d-aspartate receptor; anti-AMPA1s 1 and 2, anti-α-amino-3-hydroxy-5-methyl-4-isoxazolepropionic acid receptors 1 and 2; CASPR C, contact associated protein 2-CASPR; VGKC, voltage-gated potassium channel associated, Leucine rich glioma – inactivated protein 1/VGKC associated; GABA, gamma-aminobutyric acid B1, B2 antibodies. Anti-nuclear antibodies profile (ANA profile): p-ANCA, antineutrophil cytoplasmic antibodies – perinuclear; c-ANCA, cytoplasmic; nRNP-sm, small ribonucleoprotein antibodies, Sm, SS-A, SS-B, Scl-70, PM-Scl 100, Jo-1; CENP B, centromere protein; PCNA, proliferating cell nuclear antigen; dsDNA, nucleosomes, histones, ribosomal *P* – protein; AMA M2, antimitochondrial antibodies. No epileptiform abnormalities were noted on the EEG. MRI, magnetic resonance imaging; EEG, electroencephalogram; ADL, activities of daily living.

a. Neurometabolic blood work up includes the following list of biochemical investigations: urine screen for abnormal metabolites (ferric chloride test, DNPH test, cyanide nitroprusside test, Benedict's test, spot test for mucopolysaccharidoses), tandem mass spectrometry for blood amino acids, free carnitine, short-, medium- and long-chain acylcarnitine (glycine, alanine, valine, leucine/isoleucine, phenylalanine, tyrosine, methionine, citrulline, ornithine, arginine, proline, glutamine/lysine, glutamic acid, carnitine panel, free carnitine, C0, Acetylcarnitine, C2, Propionylcarnitine, C3, Malonylcarnitine/3-OH-butyrylcarnitine, C3DC/C4-OH, Butyrylcarnitine, C4, Methylmalonylcarnitine/ 3-OH-isovalerylcarnitine, C4DC/C5-OH, Isovaleryl/2-methylbutyrylcarnitine, C5, Tiglylcarnitine, C5:1,Glutarylcarnitine/3-OH-hexanoylcarnitine, C5-DC/C6OH, Hexanoylcarnitine, C6, Adipylcarnitine, C6DC, Octanoylcarnitine, C8, Octenoylcarnitine, C8:1, Decanoylcarnitine, C10, Decenoylcarnitine, C10:1, Decadienoylcarnitine, C10:2, Dodecanoylcarnitine, C12, Dodecenoylcarnitine, C12:1, Tetradecanoylcarnitine, C14, Tetradecenoylcarnitine, C14:1, Tetradecadienoylcarnitine, C14:2, 3-OH-tetradecanoylcarnitine, C14-OH), plasma lactate, ammonia, homocysteine, vitamin B12, aryl sulfatase and hemogram.

#### Case 1

A 6-year-old boy presented with a 3-month history of subacute onset characterised by decreased speech and interaction, decreased normative play and apparent loss of pre-existing ability to read or write. Brief periods of talking to self, irritability, crying spells and fearfulness without any apparent triggers, along with intermittent repetitive behaviours like touching ears, licking lips and insisting things be placed in a particular manner, were noted. He required assistance for previously independent activities such as toileting, bathing and feeding. There was a family history of psychotic illness and suicide in a third-degree relative.

#### Case 2

An 8-year-old girl presented with a 2-year history of subacute onset, progressive course characterised by decreased interaction and self-absorbed behaviour, followed by disorganised behaviours like disrobing herself, urinating and defecating at inappropriate places and chewing non-edible items. There was decreased meaningful interactions and cognitive abilities noted. Repeated hand washing, arranging her possessions in specific patterns, not allowing anyone to touch her belongings, and anger outbursts and aggression on non-adherence was noted in the later course. Bowel and bladder incontinence persisted for a predominant period of the illness course.

#### Case 3

A 9-year-old girl with well-adjusted pre-regression adaptive functioning except for minor difficulties in academics, presented with subacute onset, continuous course of fearfulness, crying spells and clinging to mother, followed by self-absorbed behaviour. There was loss of contextual and meaningful speech. Occasionally, she would utter the word ‘snake’ and point at her stomach, and was guarded about anyone touching her abdomen. There was further decline in her academic abilities and she needed assistance for all of her self-care activities, including toileting. She also engaged in certain repetitive behaviours like tugging on her skirt repeatedly, or repeated pacing about. There was a family history of schizophrenia in a third-degree relative.

#### Case 4

A 7-year-old boy presented with subacute onset of talking and smiling to self and decreased interaction with family members, and fearfulness resulting in unprovoked aggression toward the family members for the past month. He also had certain repetitive behaviour like pouring water on his head multiple times intermittently. It was associated with loss of functional speech and play, decline in academics abilities and need for complete assistance for toileting, dressing and bathing activities.

#### Case 5

A 10-year-old boy with mild language delay with catch up at age 5 years, presented with a 1-year history of insidious onset of decreased speech and language use associated with decreased cognitive abilities. He was found to be mostly self-absorbed, with significant reduction in both expressive and receptive language abilities almost amounting to being non-verbal. He also had decreased socio-emotional reciprocity, with increased engagement in stereotypical and self-absorbed play. There were occasional episodes of crying spells, and clinging to mother. He was completely dependent for his dressing and feeding needs, and toileting indicative of bowel and bladder incontinence. There was a history of cerebral palsy in a sibling.

#### Case 6

A 10-year-old girl presented with a 1-year history of insidious onset, progressive course characterised by initial obsessive–compulsive symptoms and fearfulness, followed by progressive social withdrawal, and decline in speech to only a few words and need-based communication. Bowel and bladder incontinence, and decreased self-care, evolved during the illness course. She had fleeting eye contact and often engaged in odd non-purposeful behaviours, stereotypic movements and utilisation behaviours. Unprovoked aggression and increased activity levels were noted since illness onset. There was a family history of schizophrenia in the father.

#### Case 7

A 7-year-old girl presented with insidious onset of loss of previously attained social and speech milestones over 6 months, along with poor eye contact, poor response to name call and not verbalising/indicating for her basic needs. She was noted to have decline in peer interactions and predominantly displayed restricted and non-functional play, with no meaningful engagement in other activities. She also had bowel and bladder incontinence over the illness course.

#### Case 8

A 6-year-old boy presented with insidious onset of loss of attained milestones in speech, social and cognitive domains. He was noted to be self-muttering with brief utterances about dinosaurs, cartoon characters or ghosts, with no further elaboration. He was also noted to have multiple stereotypical behaviours with no engagement in meaningful play, reading or writing activities. There was associated increased activity levels, sleep disturbances and contextual anger outbursts. Bowel and bladder incontinence evolved during the later course of illness.

#### Case 9

A 10-year-old boy presented with a 2-year history of insidious onset, characterised by regression of attained milestones in the language and social domains, fearfulness and crying spells. He would make brief utterances and incomprehensible sounds. Play engagement was inconsistent and rarely functional. Non-targeted motoric hyperactivity was noted. There is history of exposure to conflicts and physical abuse between parents from formative years.

#### Case 10

A 10-year-old boy presented with a 1-year duration of subacute onset of reduction in socio-communication and language amounting to almost mutism. He was noted to have atypical use of non-verbal gestures and play behaviours that were stereotypical and non-functional. Cognitive decline was evident as he could not engage in foundational academics, whereas he was above-average in grade 5 before illness onset. There was a history of parental separation and depression in his mother. There was no bowel or bladder incontinence noted.

## Discussion

All ten children presented with similar profiles of developmental regression in speech, social and cognitive milestones, and loss of adaptive skills.

### Is this autistic regression?

The onset of developmental regression was after 6 years of age, which was unlike that observed in autism.^17,18^ Two regression patterns have been described in ASD, one initially exhibiting typical socio-communicative development followed by gradual regression over the first 2 years of life, and a second pattern involving socio-communication deficits before the regression.^21^ Some longitudinal studies have reported ASD being acquired at a later age; however, it must be noted that this cohort was referred for developmental concerns at an earlier age, but only at a later date were they formally diagnosed with ASD, representing late diagnosis rather than late onset.^26^ The cases in the current study had typical development before the onset, later age at onset of regression and involved broader domains than typically seen in ASD, such as loss of adaptive skills and socio-emotional, language and cognitive regression, thus ruling out the possibility of ASD.^25,27,28^

### Do the symptom dimensions point toward a diagnosis of COS or severe depression?

All children presented with subacute/insidious onset of developmental regression and had symptoms suggestive of hallucinatory experiences at some point during the disease course. The interpretation of hallucinatory experiences was made based on serial behavioural observations. Eight children were noted to have repetitive utterances (incomprehensible phrases, names of cartoon characters), gesturing or engaging in stereotypical or non-purposeful play. Since hallucinations are symptoms elicited based on internal experiences, it becomes difficult to reliably diagnose this in young children, especially when they also have regression in socio-communication.^29^ Similarly, thought phenomena (including delusions and depressive cognitions) could not be elicited with certainty in any of our cases.

It may be challenging to delineate the psychotic phenomenon (disorganisation/hallucinations) from ritualistic or repetitive behaviours noted in ASD or CDD. ‘Disorganisation’ of thought and behaviour is one of the diagnostic criteria for schizophrenia as per the DSM-5.^30^ Disorganised thinking refers to fragmented or incoherent speech, jumping from one idea to another without logical connections and neologisms, and disorganised behaviour refers to bizarre and unexplained behaviour, including disorganisation in carrying out routine activities.^31^ Rather than disorganisation of thought and speech and psychomotor retardation seen in depression and catatonia, actual loss of functional speech associated with atypicality in socio-emotional reciprocity, and not a mere decrease, was noted in all cases. All ten children, exhibited self-absorbed behaviours associated with decreased ability to speak and comprehend, and all of them had gross loss of independence in carrying out their daily activities. These behaviours may be conceptually considered ‘disorganised behaviours’ or ‘psychomotor retardation’ under the diagnostic criteria, but phenomenologically also represent regression or loss of skills and milestones the child had previously attained.

Loss of previously acquired bowel and bladder control has been reported in about 75% of cases of CDD across multiple cohorts,^21,24^ and was present in eight out of ten of our cases. Whether this behaviour is a result of loss of previously attained milestones or the result of a new-onset psychotic phenomenon is only a matter of speculation. Some literature hints at a potential link between urinary incontinence in the initial episode of psychosis and the negative symptom dimension of schizophrenia, indicating a possible connection to underlying prefrontal cortical dysfunction. However, there is currently no definitive evidence establishing this association.^32^

Several researchers in the past have reported the diagnostic dilemma in differentiating late-onset autism, CDD and COS.^27,28^ These diagnostic entities often follow a somewhat similar course, i.e. initial neurotypical development, followed by a sudden deterioration of socio-communication skills, which may be combined with a decline in cognition, intelligence and abilities to independently carry out developmental tasks.^27^ Similar neuropsychiatric presentations may be seen in disorders such as anti-NMDA encephalitis, and neurometabolic and certain neurodegenerative disorders. Social withdrawal, hallucinatory behaviours, emotional and behavioural symptoms like anger outbursts, screaming, fearfulness associated with speech and/or movement disturbance might be one of the initial presentations in anti-NMDA encephalitis.^33^ In the current study, all ten children were evaluated by paediatric neurologists, and neurological causes such as inborn errors of metabolism, storage disorders, epilepsy, structural pathology of the central nervous system and autoimmune encephalitis were ruled out (Table 2). None of the children had seizures, focal/abnormal neurological signs or sensory impairment, as per comprehensive clinical evaluation.

Children were empirically initiated on atypical antipsychotics, as possible psychotic phenomenon were present in all cases. There was improvement in self-muttering and social engagement. Few children responded with an antipsychotic dose of <0.5–3 mg of risperidone (cases 1, 2 and 4), one child needed to switch antipsychotics (case 3) and one needed a combination of psychotropics owing to minimal response with one antipsychotic (case 6). The rationale for pharmacotherapy was largely symptom-targeted, addressing fearfulness, aggression and possible hallucinations. Selective serotonin reuptake inhibitors were considered in six patients, with the target symptom domains being anxiety and repetitive behaviours. For one child (case 3), a diagnosis of obsessive–compulsive disorder could be considered with certainty, because of repetitive behaviours predominantly in the context of repeated hand washing and preoccupations with cleanliness. The dose titration was done gradually, with careful monitoring for response and emerging side-effects. Most children tolerated the antipsychotic dose well. Flat affect, lethargy and incontinence were noted at presentation, before initiation of medications, and some of these symptoms in fact improved with antipsychotic treatment. Extrapyramidal symptoms when present were treated with anticholinergic or dose reduction of antipsychotics as required.

### Interventions and outcomes

Irrespective of the diagnostic conundrum, the interventions were formulated from a developmental lens, using principles of naturalistic developmental behavioural interventions, with active parental involvement as ‘co-therapists’ in skill building and retraining.^34^ The impact of regression on parental stress and coping was intense. A multidisciplinary team provided supportive interventions to address parental stress and to encourage continued engagement in therapeutic interventions.^4^ Initiating developmentally appropriate activities was extremely important.^4,21^ Developmentally appropriate tasks were reintroduced through play and structured activities, and graded re-entry to school was planned based on developmental readiness.

All children appeared to follow a static course after the period of regression. The response to treatment was gradual over 6 months to 1 year, in the domains of repetitive and hallucinatory behaviours, aggression, irritability and lesser dependence for activities of daily living. Although eight out of ten children presented with loss of bowel/bladder control during peak of the illness course, it resolved as they showed improvement in other domains. Some improvement was noted in speech and language in seven children. Residual symptoms were noted in all children at 1-year follow-up, in the domains of not attaining age-appropriate speech and language, socio-emotional reciprocity and cognitive abilities, and nine out of ten children did not attain pre-regression functioning (Table 2). Similar outcomes were reported in previous studies.^24,25^

Developmental regression in the absence of identifiable neurological causes is one of the enigmatic presentations in child psychiatry. Based on longitudinal evaluation of phenomenology, course and outcome at 1 year, COS may be a possible diagnosis for cases 1, 3, 4, 6 and 8; however, the bowel and bladder incontinence preclude the same. Cases 2, 5, 7, 9 and 10 could not be placed under the diagnostic category of schizophrenia, depression or late-onset autism.

Diagnostic conundrums when seen cross-sectionally may be a challenge. In such cases, the child and adolescent mental health clinician's responsibility and immediate aim is to get corroborative evidence for premorbid functioning and extensive aetiological evaluation. For severe manifestations such as developmental regression, where the illness is still evolving, considering CDD as a non-etiological and transitory/tentative diagnosis would aid against premature diagnostic categorisation and provide scope for ongoing aetiological search. Developmental regression cuts across multiple psychiatric and neurological disorders among children. Persistence of deficits in language and socio-emotional development over the course of illness, despite treatment, may raise the possibility of CDD. Over time, children may move out of CDD toward other diagnoses, or may continue to be categorised as CDD if disorganisation and/or loss of acquired skills persist.

To the authors knowledge, this is one of the few large case series with longitudinal follow-up of course and outcome of late-onset developmental regression. Although aetiological work-up was exhaustive, molecular genetics studies and serial neuroimaging could have provided critical information. The team aims to attempt the same at future follow-ups. Irrespective of the diagnostic challenges, transdiagnostic and dimensional framework to interventions is a recommendation for best clinical practice. Long-term longitudinal evaluation is required for these subset of children to understand the transitions in phenomenology, course and outcome, and will provide critical insights on biological underpinnings of developmental regression.

## Supporting information

Abraham et al. supplementary material 1 Abraham et al. supplementary material

Abraham et al. supplementary material 2 Abraham et al. supplementary material

Abraham et al. supplementary material 3 Abraham et al. supplementary material

## Data Availability

The data that support the findings of this study are available from the corresponding author, H.M., upon reasonable request.
