# Supplementary material for: The diagnostic conundrum of late-onset developmental regression in child psychiatry: case series
Source: BJPsych Open. 2025 Jan 27;11(1):e25. doi: 10.1192/bjo.2024.840 (PMC11823004; doi:10.1192/bjo.2024.840)
Supplement: Abraham et al. supplementary material 1 — Abraham et al. supplementary material [file S2056472424008408sup001.docx]

**Patient Consent Form**

**Name of the patient:**

**Name of the Legally Authorized Representative (parent/guardian):**

**Relationship:**

**Provisional title of the article in which the material will be included:**

**Consent:**

I________________________________________, parent of ______________________________, give my consent for the clinical material about my child to appear in a scientific publication. I have been informed about the nature of the scientific and academic writing related to child’s case.

I understand the following:

(1) The Material will be published without any identifying details (my/the patient’s name attached). (2) The Material may show or include details of my/the patient’s medical condition or injury and any prognosis, treatment or surgery that I have/the patient has, had or may have in the future. I understand that my name and initials will not be published and that efforts will be made to conceal my identity, and that anonymity will be ensured. I also understand that no images will be included in this publication. (3) The article may be published in a journal which is distributed worldwide. The publication will be accessible to doctors and other healthcare professionals but are also seen by many others including academics, students and journalists. (4) I/the patient will not receive any financial benefit from publication of the article.

I have understood that I have the right to refuse my consent and it will not adversely affect my treatment.

Signature of the parent/ guardian:

Relationship with the patient (child):

Signature of witness:

Signature of the Doctor/ investigator:

Date: Place:
