# Supplementary material for: The diagnostic conundrum of late-onset developmental regression in child psychiatry: case series
Source: BJPsych Open. 2025 Jan 27;11(1):e25. doi: 10.1192/bjo.2024.840 (PMC11823004; doi:10.1192/bjo.2024.840)
Supplement: Abraham et al. supplementary material 2 — Abraham et al. supplementary material [file S2056472424008408sup002.pdf]

NATIONAL INSTITUTE OF MENTAL HEALTH AND NEURO SCIENCES
(INSTITUTE OF NATIONAL IMPORTANCE)
P.B. NO.2900, HOSUR ROAD, BENGALURU - 560 029 (INDIA)

Dr. Prabha S Chandra
Sr. Professor of Psychiatry
Dean & Member Secretary
Ethics Committee (Behavioural Science Division)

Off : 26995004
Fax: 91-80-26564830/26566811
email:deannimhans@yahoo.com

No. NIMHANS/EC (BEH.SC.DIV.)MEETNG/2024 Date: 13.02.2024

1.	**Name of the investigator:**	Dr. Harshini M, Assistant Professor, Department of Child and Adolescent Psychiatry
2.	**Reference number of the investigator:**	Letter dated : 03.02.2024
3.	**Title of the proposal submitted for ethical clearance:**	Clinical course and outcome of children presenting with developmental regression: A record review.
4.	**Nature of the proposal submitted for ethical clearance:**	Non Funded Research Project
5.	**New Review** ✓ **Revised Review**	**Expedited Review**
6.	**Date of Review by the Member Secretary:**	13.02.2024
7.	**Date of previous review, if revised application:**	-

Decision: Approved.

Dr. Prabha S Chandra
Dean (Behavioural Science) & Member Secretary
IEC (BEHAVIOURAL SCIENCES DIVISION)

Ole
