# Supplementary material for: The diagnostic conundrum of late-onset developmental regression in child psychiatry: case series
Source: BJPsych Open. 2025 Jan 27;11(1):e25. doi: 10.1192/bjo.2024.840 (PMC11823004; doi:10.1192/bjo.2024.840)
Supplement: Abraham et al. supplementary material 3 — Abraham et al. supplementary material [file S2056472424008408sup003.pdf]

NATIONAL INSTITUTE OF MENTAL HEALTH AND NEUROSCIENCES
(INSTITUTE OF NATIONAL IMPORTANCE), BANGALORE – 560 029

03.02.2024

From:
Dr. Harshini. M
Assistant Professor,
Department of Child and Adolescent Psychiatry
NIMHANS

DIRECTOR'S SECRETARIAT
RECEIVED ON
06 FEB 2024
NIMHANS, BENGALURU-29

To:
Chairperson/Member Secretary,
NIMHANS Ethics Committee (Behavioural Sciences Division)
National Institute of Mental Health and Neurosciences
Bangalore-560029

"Through Proper Channel"

Respected Sir/Mam,

Sub: Ethical clearance for a non-funded study titled **'Clinical course and outcome of children presenting with developmental regression: A record review'**

I am herewith submitting the research protocol for the non-funded study titled **'Clinical course and outcome of children presenting with developmental regression: A record review'** for consideration by the Institute Ethics Committee.

This is a non-funded study involving clinical details from the hospital records. No patients will be contacted for the study purpose.

We request for an **expedited review.**

Thanking you,
Yours sincerely,

Dr.Harshini. M

Date: 03/02/2024

The proposed ~~retrospective~~ chart review has been discussed in the Department faculty meeting and was found to have adequate scientific merit.
I recommend an expedited review and ethical approval of this proposal
06/02/2024.

डॉ. के जॉन विजय सागर
Dr. K. John Vijay Sagar
अनुकमांक/Reg. No. TMN/20010001483/KTK
प्राध्यापक और प्रमुख/Professor and Head
बाल और किशोर मनश्चिकित्सा विभाग
Dept. of Child & Adolescent Psychiatry

CAP

Check List of Documents
(Required to be submitted to Ethics Committee)

**Documents** (1+1 Hard Copy + PDF e-version by e-mail with all the duly filled in IEC formats including covering letter)	**Remarks** (Tick the appropriate)
1. Covering letter for the project proposal ("Through Proper Channel"	Yes
2. Summary Sheet of the Protocol	Yes
3. Research Project proposal submitted for Ethical Clearance as per the NIMHANS Ethics Committee guidelines	Yes
i. Consent form duly signed by the investigator/collaborators. (Duly signed Attestation & Declaration Form)	Yes
**ii.** Consent of the concerned Head of the department	Yes
**iii.** Duly signed Attestation & Declaration Form	Yes
iv. Authorization/ sanctioning letter (finance sanction) from sponsoring agency	NA
v. Informed Consent form as per the guidelines of NIMHANS IEC	NA
vi. Consent form for carrying out the required investigations from the concerned Heads of Department(s). if applicable......	NA
vii. Undertaking by the investigator(s)	Yes

Dharshini

डॉ. हर्षिनी .एम/Dr. Harshini M
अनुक्रमांक/Reg. No. KMC 201400013[illegible] K
सहायक प्राध्यापक/Assistant Professor
बाल और किशोर मनश्चिकित्सा विभाग
Dept. of Child & Adolescent Psychiatry
निम्हान्स, बेंगलूरु/NIMHANS, Bengaluru-29

Dr Harshini. M
Assistant Professor,
Department of Child and Adolescent Psychiatry,
NIMHANS.

SUMMARY SHEET OF THE RESEARCH PROJECT SUBMITTED TO THE HUMAN ETHICS COMMITTEE (BEHAVIOURAL SCIENCES) OF NIMHANS

1	Title and duration of the project	Clinical course and outcome of children presenting with developmental regression: A record review	
2	Investigators and their Department(s)	**Principal Investigator:**<br><br>Dr. Harshini Manohar<br>Assistant Professor,<br>Department of Child and Adolescent Psychiatry,<br>National Institute of Mental Health and Neurosciences.	
3	Funded or non-funded project? If yes, name of the funding agency (Govt, Private, Foreign)<br>OR<br>Is the project being submitted for funding?	Non funded	
4	Are human subjects involved in the study? IF yes, mention type of participants (patients, relatives, professionals etc.	No (details from clinical case records and hospital files will be collected for the study).	
5	Does the study involve healthy volunteers?	No	
6	Does the study involve vulnerable population (Children, Pregnant women, personas with disabilities, persons with mental illness etc.)?	No (details from clinical case records and hospital files will be collected for the study).	
7	Study Design (Describe briefly)	Record review	
8	Is the submission in NIMHANS – IEC format? (should not be in the format of the funding agency)	Yes	
9	Procedures and risks: list the procedures carried out and the possible risk (classified as less than minimal, minimal, low or high)	Procedures<br>NA	Risk<br>NA
10	Detail the measures taken for reducing the risk	NA	
11	Does the study involve biological specimens? If yes, list the type specimens and the amount	No	
12	Does the research / study involve:	NA	

	• Human exposure to radioactive agents?<br>• Human exposure to infectious agents?<br>• Investigational new drug?<br>• Investigational new device?<br>• New treatment regime?<br>• Use of new vaccines?<br>• Observation of public behavior?<br>• Fetal tissue or abortus?<br>• Pathological or diagnostic clinical specimen only? (Mention source............)<br>• Existing data available via public archives source? (Specify....................;...)<br>• Existing data available from co-investigator?	No<br>No<br>No<br>No<br>No<br>No<br>No<br><br>No<br><br>No
13	Is informed consent form attached? If not, mention justification	No (details from clinical case records and hospital files will be collected for the study). No patients will be contacted for the study purpose.
14	Does the informed consent form address the following :	NA
	a) Provide adequate information about title, purpose, procedures, & details of participation in a layperson's language to the participant?<br>b) Is the method of selection of subjects including random selection, if applicable is explained?<br>c) Are procedures including invasive procedures and possible risks adequately explained?<br>d) Are financial implications explained to the patient/legal guardian?<br>e) Are there separate ICF's for participants, legally acceptable representatives (LAR'S) and volunteers? If so, list<br>f) If subject is a minor, is there an appropriate assent form?<br>g) Is the course of action, in case, any abnormalities are detected during the investigation, clearly spelt out?<br>h) Is provision for the subject to opt out of the study made explicitly<br>i) Is confidentiality of the subject's data assured<br>j) If major risks are involved, is mechanism of treatment / compensation for any injury suffered (e.g., Insurance) clearly spelt out?<br>k) Are the contact details of the PI and investigators provided?	

	l) If the study involves a biological specimen, is the consent obtained only for the current study or for future research also?<br>m) Are the details of payments included?	
15	In case of Clinical Trials	NA (Not a trial)
	a) Name of the drug (device) being investigated?	
	b) Is the product currently in clinical use in India?	
	c) Does the trial involve an investigational new drug	
	d) Name and address of the manufacturer?	
	e) Is there a DCGI approval for the trial?	
	f) Is it a multi-centric trial? If yes, how many Indian and how many foreign centres are involved?	
	g) Does the drug have statutory approval for clinical use in the country of origin?	
	h) Does the study have a placebo arm? If does, what risk does it entail for the subjects? justify the use of the placebo	
	i) Is any standard treatment withheld in any subject as a part of the study? If yes, provide justification for the same and assurance of patient safety	
	j) Are the possible risks documented in the literature adequately explained in the ICF	
	k) Is the trial covered by an insurance scheme? (If yes, details)	
	l) Are there any conflicts of interest for the investigators: e.g., remuneration paid to the investigator by the company, investigator's financial involvement in the company	
	m) Implications of costs to the patient<br>n) Does the trial cover the patient's treatment cost,<br>o) Does the trial pay for the additional costs to the patient on account of his participation in the trial?<br>p) Is the patient / volunteer provided remuneration for participating in the trial?	
16	In case of chart review / records-based studies, mention as to how the identity of the patient is delinked and how confidentiality is maintained	The patient details would be anonymized. The data would be de-linked from identification details like name, address and hospital number and assigned a unique alpha-numeric code to maintain confidentiality.
17	Does the research deal with sensitive aspects of the	No

	subject's behavior such as sexual behavior, alcohol use or illegal conduct such as drug use?<br><br>Are there any elements in the protocol that are likely to induce anxiety or distress to the subject (e.g., intrusive questionnaire, presentation of material that is unpleasant to the participant etc.)	
18	Mention specific Ethical Issues involved in the proposed research (List & briefly describe)	NA
19	Is there payment to participants? If yes, details	No
20	Whether the proposed study is a collaborative study? Yes/No<br>If yes, does the other institution have IERB? Yes/No<br>If yes, have you received that IERB approval? Yes/No	No
21	Is there a Bio-safety Department in the collaborative institute for disposing of biological samples in a scientific manner after carrying out investigation?	NA
22	Mention whether the sample size allows enough power to detect the difference/results expected from the investigation	NA
23	Is there any conflict of interest?	No
24	Is there Financial interest of (i) investigators or (ii) sponsors? If yes, provide details	No

TITLE PAGE

NATIONAL INSTITUTE OF MENTAL HEALTH AND NEUROSCIENCES

TITLE: 'Clinical course and outcome of children presenting with developmental regression: A record review'

Consent of the guide, co-guide/ joint guide and Head of the Department

Principal Investigator:

Dr. Harshini M,
Assistant Professor,
Department of Child and Adolescent Psychiatry,
NIMHANS.

डॉ. हर्षिनी .एम/Dr. Harshini .M
अनुक्रमांक/Reg. No. KMC 20140001382KTK
सहायक प्राध्यापक/Assistant Professor
बाल और किशोर मनश्चिकित्सा विभाग
Dept. of Child & Adolescent Psychiatry
निम्हान्स, बेंगलूरु/NIMHANS, Bengaluru-29

Head of Department:

Prof. Dr. John Vijaysagar Kommu,
Professor and Head,
Department of Child and Adolescent Psychiatry,
NIMHANS

डॉ. के जॉन विजय सागर
Dr. K. John Vijay Sagar
अनुक्रमांक/Reg. No. TMN/20010001483/KT[illegible]
प्राध्यापक और प्रमुख/Professor and Hea[illegible]
बाल और किशोर मनश्चिकित्सा विभा[illegible]
Dept. of Child & Adolescent Psychiatry
निम्हान्स/NIMHANS, बेंगलूरु/Bengaluru-29

DECLARATION AND ATTESTATION FORM

I. I/We have read the terms and conditions for.................NA........................ (insert name of the funding agency/sponsoring agency) Research Grant. Necessary Institutional facilities will be provided, if the research project is approved for financial assistance.

II. I/We agree to submit within one month from the date of termination of the project, the final report and a list of articles, both expendable and non-expendable, left on the closure of the project.

III. I/We agree to submit audited statement of accounts duly signed by the auditors of the Institute.

Signature of the:-

a) **Principal Investigator (with date)** Harshini

Dr. Harshini. M

Seal: डॉ. हर्षिनी .एम/Dr. Harshini .M
अनुक्रमांक/Reg. No. KMC 20140001382KTK
सहायक प्राध्यापक/Assistant Professor
बाल और किशोर मनश्चिकित्सा विभाग
Dept. of Child & Adolescent Psychiatry
निम्हान्स, बेंगलूरु/NIMHANS, Bengaluru-29

b) **Head of the department with remarks (with date)** [signature]

Name in Capital Letters: DR. K. JOHN VIJAY SAGAR

Seal: डॉ. के जॉन विजय सागर
Dr. K. John Vijay Sagar
अनुक्रमांक/Reg. No. TMN/20010001483/KTK
प्राध्यापक और प्रमुख/Professor and Head
बाल और किशोर मनश्चिकित्सा विभाग
Dept. of Child & Adolescent Psychiatry
निम्हान्स/NIMHANS, बेंगलूरु/Bengaluru-29

d) **Head of the Institution with remarks:** [signature]

DIRECTOR i/c
National Institute of Mental
Health and Neuro Sciences, Bengaluru - 560 029

Name in Capital Letters:

Seal:

Date: 6.2.24

Research proposal:

Title: 'Clinical course and outcome of children presenting with developmental regression: A record review'

Review of Literature and need for the study:

The progressive loss of previously acquired developmental milestones and competencies is a concerning clinical picture in child psychiatry and neurology, posing diagnostic and intervention challenges. It may suggest an underlying neurological aetiology denoted by the term *'progressive intellectual and neurological deterioration'.(1)* There is evidence for developmental regression, particularly early regression during the first 2 years of life in autism spectrum disorder (ASD), epileptic encephalopathies, genetic syndromes such as Rett's syndrome and Phelan McDermid syndrome, and late regression in Down syndrome.(2–4)

Apparent developmental and behavioural regression is observed in childhood-onset schizophrenia (COS),(5–7) and post-traumatic stress disorder(1,8), though there is a paucity of systematic literature on this phenomena.(6) The National Institute of Mental Health (NIMH) COS longitudinal study, the largest study of COS to date, reported a prevalence of 0.04%.(9–11) COS (onset <13 years) is rare in occurrence, and symptom dimensions include both positive and negative symptoms, with disorganisation being less common.(12) Developmental deviance, especially in the speech and language domains, and poor premorbid adjustment before illness onset has been reported.(13,14) The diagnosis of psychotic symptoms in children is often complex owing to the symptomatic overlap with other emotional, behavioural and neurodevelopmental disorders (NDDs). When psychotic symptoms are associated with developmental regression, the diagnostic dilemma becomes far more pronounced.

Regression in the socio-communication domain is commonly seen in ASD, termed 'autistic regression'.(15) Meta-analytic reviews report prevalence rates of 30-32.2 % of regression in ASD, with an average age of onset of 19.8 months.(16,17) Prospective designs may have better potential to capture more subtle skill loss, particularly early socio-communication behaviours, compared to retrospective studies.(18)

The phenomenon of 'autistic regression' does not explain the later age of onset of regression, which occurs in a subset of children. Theodore Heller first described this phenomenon *'dementia infantalis'* in 6 previously normal children who presented with developmental regression.(19) In the International Classification of Diseases, 10th Revision and Diagnostic and Statistical Manual of Mental Disorders, Fourth Edition, Text Revision, the disorder was termed childhood disintegrative disorder (CDD).(19) Misdiagnosis of CDD as COS, potentially due to severe social impairment and withdrawn behaviour accompanied by stereotypic movements resembling symptoms of psychosis have been reported.(20) Also, psychotic symptoms were observed in about 33% of children who received a diagnosis of CDD.(19)

With the advent of technology, causes for later onset developmental regression, such as autoimmune encephalitis, neurometabolic, and neurodegenerative disorders were increasingly identified. CDD as a distinct diagnosis has been a subject of debate due to its shared characteristics with ASD, such as core socio-communication impairments, comorbid intellectual disability (ID), and epilepsy; thus, CDD was subsumed under ASD. Though the

debate on the diagnostic validity continues, CDD does have features supporting it to be distinct from ASD.(21–23) Despite increasing scientific and clinical interest, developmental regression continues to pose multiple challenges. Atypical to what is currently understood about this phenomenon, a subset of children continue to present with later onset regression in the background of neurotypical development, with no identifiable neurological causes.

Aim and objectives:

The aim of this study is to understand the course and outcome of children presenting with clinically significant loss of previously acquired skills (developmental regression) with no identifiable neurological causes.

Objectives:

1. To describe the clinical characteristics of children presented with developmental regression.
2. To study the course and outcome of the illness and developmental trajectory.

Methods:

Study design: Record review (of prospective clinical follow-up).

Setting: The study will be conducted at the Dept. of Child and Adolescent psychiatry, National Institute of Mental Health and Neurosciences (NIMHANS), Bengaluru, India. Medical records of children meeting the inclusion criteria will be reviewed in detail.

Study participants: Children with clinically significant loss of previously acquired skills (developmental regression), presenting to the inpatient/outpatient department of child and adolescent psychiatry, with no identifiable neurological causes on extensive evaluation.

Inclusion Criteria:

1. Children with clinically significant loss of previously acquired skills in more than two developmental domains (speech, language, cognitive, socio-emotional, adaptive skills and play).
2. Normal development prior to regression.
3. Onset of regression more than 6 years of age.
4. No identifiable neurological causes on clinical evaluation or investigations.
5. Clinical follow-up at NIMHANS up to 1 year.

Exclusion Criteria:

1. Children with regression at an earlier age.
2. Identifiable neurological causes/incomplete organic evaluation.

Study duration: 5 years.

Study procedure:

Case files of children registered under CAP, fulfilling the eligibility criteria during the period January 2019- December 2023 will be reviewed.

Prospectively, children fulfilling the eligibility criteria will be included over the period of next 5 years. Clinical histories, details of investigations, course and outcome will be collected from the patient files. Routine clinical follow-up details will be collected.

Based on best-practice guidelines, all children presenting with clinically significant loss of acquired skills undergo extensive investigations including EEG, MRI Brain, neurometabolic panel, autoimmune panel to rule out organic causes (14,22). Details of the same will be collected from patient files.

The details from the medical records will be entered into a standardized semi-structured data collection proforma.

Measures:

Sample size: The case files of all children fulfilling the eligibility criteria will be included. Since this is a rare clinical presentation, a pre-determined sample size cannot be estimated.

Statistical analysis: Descriptive statistics will be used to describe the cohort characteristics. Association of the demographic and independent variables and dependent variables will be analyzed.

Expected outcomes and implications of the study:

1. The study will provide insights on clinical course and outcome of children presenting with developmental regression, a challenging presentation in child psychiatry.
2. Insights for future studies to understand the neurobiological underpinnings of the phenomenon.

Ethical issues:

1. The research protocol will be implemented in strict adherence to the National Ethical Guidelines for Biomedical and Health Research involving human participants (2017) by the Indian Council of Medical Research.
2. As per standard guidelines, approval from the Institute Ethics Committee will be obtained prior to beginning of the study.
3. Deidentified clinical data will be collected from the hospital records. Clinical follow-up details will be collected from the files. Confidentiality of information will be maintained throughout the study. Participants will not be contacted for the study purpose. This study does not involve any risk to the participants.
4. Deidentified data will be stored in a password protected computer and will be accessible only by the principal and co-investigators of the proposed study. The data will be stored for 3 years after completion of the study.

References:

1. Holland J, Brown R. Developmental regression: assessment and investigation. Paediatr Child Health [Internet]. 2017 [cited 2024 Jan 27];27(6):253–9. Available from: https://www.sciencedirect.com/science/article/pii/S1751722217300136

2. Zhang D, Bedogni F, Boterberg S, Camfield C, Camfield P, Charman T, et al. Towards a consensus on developmental regression. Neurosci Biobehav Rev [Internet]. 2019 Dec [cited 2023 May 28];107:3–5. Available from: https://linkinghub.elsevier.com/retrieve/pii/S0149763419307110

3. Bonne S, Iftimovici A, Mircher C, Conte M, Louveau C, Legrand A, et al. Down syndrome regression disorder, a case series: Clinical characterization and therapeutic approaches. Front Neurosci [Internet]. 2023 [cited 2024 Jan 27];17:1126973. Available from: https://www.frontiersin.org/articles/10.3389/fnins.2023.1126973/full

4. Furley K, Mehra C, RP GK, Fahey MC, Hunter MF, Williams K, et al. Developmental Regression in Children: Current and Future Directions. Cortex [Internet]. 2023 [cited 2024 Feb 2]; Available from: https://www.sciencedirect.com/science/article/pii/S0010945223002186

5. Slomiak S, Matalon DR, Roth L. Very Early-Onset Schizophrenia in a Six-Year-Old Boy. Am J Psychiatry Resid J [Internet]. 2017 Feb [cited 2024 Jan 27];12(2):9–11. Available from: http://psychiatryonline.org/doi/10.1176/appi.ajp-rj.2017.120204

6. Aneja J, Singhai K, Paul K. Very early-onset psychosis/schizophrenia: case studies of spectrum of presentation and management issues. J Fam Med Prim Care [Internet]. 2018 [cited 2024 Jan 27];7(6):1566. Available from: https://www.ncbi.nlm.nih.gov/pmc/articles/PMC6293945/

7. Jha A, Kunwar A, Dhonju G. Childhood Onset Psychosis: A Case Report. J Psychiatr Assoc Nepal. 2020;9(1):64–5.

8. Feldman R, Vengrober A. Posttraumatic Stress Disorder in Infants and Young Children Exposed to War-Related Trauma. J Am Acad Child Adolesc Psychiatry [Internet]. 2011 Jul 1 [cited 2024 Feb 3];50(7):645–58. Available from: https://www.sciencedirect.com/science/article/pii/S0890856711001997

9. Fernandez A, Drozd MM, Thümmler S, Dor E, Capovilla M, Askenazy F, et al. Childhood-onset schizophrenia: a systematic overview of its genetic heterogeneity from classical studies to the genomic era. Front Genet [Internet]. 2019 [cited 2024 Jan 27];10:1137. Available from: https://www.frontiersin.org/articles/10.3389/fgene.2019.01137/full

10. Gochman P, Miller R, Rapoport JL. Childhood-Onset Schizophrenia: The Challenge of Diagnosis. Curr Psychiatry Rep [Internet]. 2011 Oct [cited 2024 Jan 27];13(5):321–2. Available from: http://link.springer.com/10.1007/s11920-011-0212-4

11. Gordon CT, Frazier JA, McKenna K, Giedd J, Zametkin A, Kaysen D, et al. Childhood-onset schizophrenia: an NIMH study in progress. Schizophr Bull [Internet]. 1994 [cited 2024 Jan 27];20(4):697–712. Available from: https://academic.oup.com/schizophreniabulletin/article-abstract/20/4/697/1933458

12. Craddock KE, Zhou X, Liu S, Gochman P, Dickinson D, Rapoport JL. Symptom dimensions and subgroups in childhood-onset schizophrenia. Schizophr Res [Internet]. 2018 [cited 2024 Jan 27];197:71–7. Available from: https://www.sciencedirect.com/science/article/pii/S0920996417306734

13. Vourdas A, Pipe R, Corrigall R, Frangou S. Increased developmental deviance and premorbid dysfunction in early onset schizophrenia. Schizophr Res [Internet]. 2003 [cited 2024 Jan 27];62(1–2):13–22. Available from: https://www.sciencedirect.com/science/article/pii/S0920996402004292

14. Driver DI, Gogtay N, Rapoport JL. Childhood onset schizophrenia and early onset schizophrenia spectrum disorders. Child Adolesc Psychiatr Clin [Internet]. 2013 [cited 2024 Jan 27];22(4):539–55. Available from: https://www.childpsych.theclinics.com/article/S1056-4993(13)00024-2/abstract

15. Matson JL, Kozlowski AM. Autistic regression. Res Autism Spectr Disord [Internet]. 2010 [cited 2024 Jan 27];4(3):340–5. Available from: https://www.sciencedirect.com/science/article/pii/S1750946709001123

16. Barger BD, Campbell JM, McDonough JD. Prevalence and Onset of Regression within Autism Spectrum Disorders: A Meta-analytic Review. J Autism Dev Disord [Internet]. 2013 Apr [cited 2024 Jan 27];43(4):817–28. Available from: http://link.springer.com/10.1007/s10803-012-1621-x

17. Tan C, Frewer V, Cox G, Williams K, Ure A. Prevalence and Age of Onset of Regression in Children with Autism Spectrum Disorder: A Systematic Review and Meta-analytical Update. Autism Res [Internet]. 2021 [cited 2023 Jun 11];14(3):582–98. Available from: https://onlinelibrary.wiley.com/doi/abs/10.1002/aur.2463

18. Pearson N, Charman T, Happé F, Bolton PF, McEwen FS. Regression in autism spectrum disorder: Reconciling findings from retrospective and prospective research. Autism Res [Internet]. 2018 Dec [cited 2024 Jan 27];11(12):1602–20. Available from: https://onlinelibrary.wiley.com/doi/10.1002/aur.2035

19. Mehra C, Sil A, Hedderly T, Kyriakopoulos M, Lim M, Turnbull J, et al. Childhood disintegrative disorder and autism spectrum disorder: a systematic review. Dev Med Child Neurol [Internet]. 2019 May [cited 2024 Jan 27];61(5):523–34. Available from: https://onlinelibrary.wiley.com/doi/10.1111/dmcn.14126

20. Sawant NS, Parker S, Kulkarni P. Childhood disintegrative disorder misdiagnosed as childhood-onset schizophrenia. South Afr J Psychiatry [Internet]. 2014 [cited 2024 Jan 27];20(3):94–5. Available from: http://www.scielo.org.za/scielo.php?pid=S2078-67862014000300006&script=sci_arttext

21. Malhotra S, Gupta N. Childhood disintegrative disorder. Eur Child Adolesc Psychiatry [Internet]. 2002 [cited 2024 Jan 27];11(3):108. Available from: https://search.proquest.com/openview/32b5b0978274a1f64f4cee9c9c3b0ca0/1?pq-origsite=gscholar&cbl=32987

22. Rosman NP, Bergia BM. Childhood Disintegrative Disorder: Distinction From Autistic Disorder and Predictors of Outcome. J Child Neurol [Internet]. 2013 Dec [cited 2024 Jan 27];28(12):1587–98. Available from: http://journals.sagepub.com/doi/10.1177/0883073812472391

23. Volkmar FR, Cohen DJ. Disintegrative disorder or "late onset" autism. Journal of Child Psychology and Psychiatry. 1989 Sep;30(5):717-24.24.

24. Elias R, Lord C. Diagnostic stability in individuals with autism spectrum disorder: insights from a longitudinal follow-up study. J Child Psychol Psychiatry [Internet]. 2022 Sep [cited 2024 Jan 27];63(9):973–83. Available from: https://acamh.onlinelibrary.wiley.com/doi/10.1111/jcpp.13551

03-02-2024

From:
Dr. Harshini. M
Assistant Professor,
Department of Child and Adolescent Psychiatry,
NIMHANS, Bangalore – 560029

To:
Chairperson/Member-Secretary,
NIMHANS Ethics Committee,
National Institute of Mental Health and Neurosciences
Bangalore-560029

Sub: Ethical clearance for Research Project / Protocol titled **"Clinical course and outcome of children presenting with developmental regression: A retrospective review'**

UNDERTAKING

With respect to the above said Research Project involving human subjects for which the ethical clearance being sought, I am to state that I have gone through the "ICMR Ethical guidelines 2017" and am aware of the rules governing the studies involving the human subjects. I am also aware that these guidelines are strictly to be followed while carrying out the above said research project involving human subjects.

Further, I also affirm that I will be responsible to keep inform the IEC of,

i. Any serious and unexpected adverse events and remedial steps taken to tackle them.
ii. Any new information that may influence the conduct of the study.
iii. Any changes made in the consent form.
iv. In the event of need to amend the original protocol approved by the EC, the proposed amendment shall be brought to the notice of EC for its consideration and approval. Under no circumstances I/we deviate from the original approved protocol without prior consent to that effect from the IEC.

Date: 03/02/2024

Name and Signature of the Principal Investigator

Harshini

Dr. Harshini. M.
Assistant Professor.
Department of Child and Adolescent Psychiatry,
NIMHANS.

Rtf
[illegible signature]

डॉ. के जॉन विजय सागर
Dr. K. John Vijay Sagar
अनुक्रमांक/Reg. No. TMN/20010001483/KTK
प्राध्यापक और प्रमुख/Professor and Head
बाल और किशोर मनश्चिकित्सा विभाग
Dept. of Child & Adolescent Psychiatry
निम्हान्स/NIMHANS, बेंगलूरु/Bengaluru-29
